# Supplementary material for: Identification of photoperiod-induced specific miRNAs in the adrenal glands of Sunite sheep (Ovis aries)
Source: Front Vet Sci. 2022 Jul 22;9:888207. doi: 10.3389/fvets.2022.888207 (PMC9354845; doi:10.3389/fvets.2022.888207)
Supplement: Supplementary file 1 [file Data_Sheet_1.ZIP › Supplementary file/miRDeep2 file.zip!/miRDeep2 file/Know_Structure/SPLP42A1/oar-mir-758.pdf]

miRBase precursor : oar-mir-758
Total read count : 513
oar-miR-758-5p read count : 0
oar-miR-758-3p read count : 508
remaining reads : 5

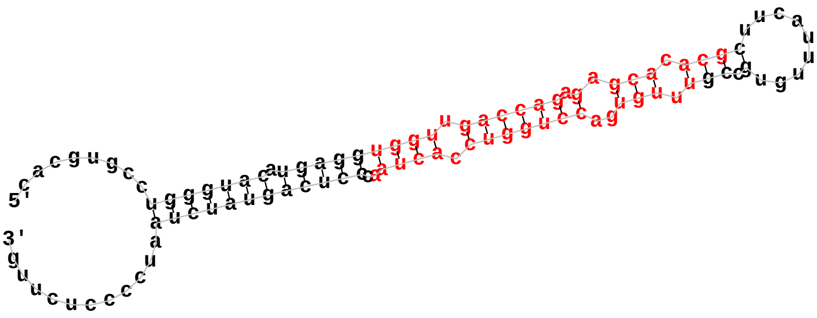

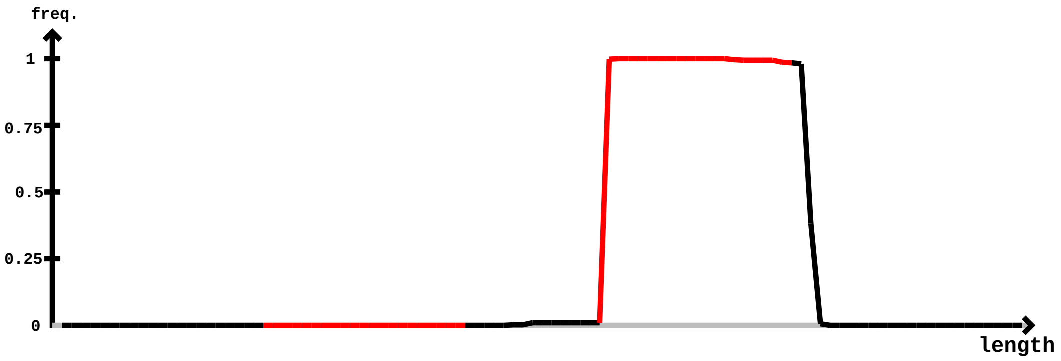


```
                                                                                                   oar-miR-758-3p
                                      oar-miR-758-5p
5'- cacgugccuggguacaugaggugguugaccagagagcacacgcuucauuugugccguuugugaccugguccacuaacccucaguaucuaauccccucuug   -3'    exp
    ........(((((((.(((((((((.((((((.(.(((.((((.........).))).)))..))))))).))))..))))))))))))...........    reads      mm         sample
    ..............................................auuugugccguuugugaccugguc..............................    1          0          seq
    ................................................uugugccguuugugaccuggu...............................    2          0          seq
    ................................................uugugccguuugugaccugguccacu..........................    2          0          seq
    ........................................................uuugugaccugguccacu..........................    2          0          seq
    ........................................................uuugugaccugguccacua.........................    1          0          seq
    ........................................................uuugugaccugguccacuaa........................    2          0          seq
    ........................................................uuugugaccugguccacuaac.......................    307        0          seq
    ........................................................uuugugaccugguccacuaacc......................    192        0          seq
    ........................................................uuugugaccugguccacuaaccc.....................    3          0          seq
    .........................................................uugugaccugguccacuaacc......................    1          0          seq
```
